# Reimplantation approach for an anomalous aortic origin of the right coronary artery with an aberrant right subclavian artery

**DOI:** 10.1186/s13019-022-02029-9

**Published:** 2022-11-07

**Authors:** Keiichiro Kasama, Yasuko Uranaka, Hiroto Tomita, Takuya Saba, Takahiro Koura, Yukio Yamashita, Shinichi Suzuki

**Affiliations:** 1 Department of Cardiovascular Surgery, Yokohama Municipal Citizens Hospital, 1-1 Mitsuzawa Nishicho, Kanagawa-ku, Yokohama, 221-0855 Japan; 2 Department of Cardiology, Yokohama Municipal Citizens Hospital, Yokohama, Japan; 3 Department of Pediatrics, Yokohama Municipal Citizens Hospital, Yokohama, Japan; 4 Department of Surgery, Yokohama City University Graduate of Medicine, Yokohama, Japan

**Keywords:** Aberrant subclavian artery, Right coronary artery, Reimplementation, Aorta, Anomaly

## Abstract

Anomalous aortic origin of the right coronary artery is a rare disease. Although there are various reports on its treatment, the method of the surgical approach is still controversial. Here, we present a rare case of a 17 year-old man who had an anomalous aortic origin of the right coronary artery with an aberrant right subclavian artery. As a treatment, he underwent reimplantation of the right coronary artery. The surgical approach for the anomalous aortic origin of the right coronary artery should be selected by considering the age of the patient and size of the right coronary artery.

## Background

Coronary artery abnormality is uncommon; however, there are various reports on its treatment. The prevalence of anomalous aortic origin of the right coronary artery (AAORCA) ranges between 0.1 and 1% [1, 2]. There are various discussions regarding the surgical indications and treatment approaches. Mainly, unroofing procedure, coronary artery bypass grafting, and reimplantation are some of the several surgical options for AAORCA. However, the gold standard for the surgical treatment remains controversial. The surgical approach should be selected by considering the age of the patient and size of the right coronary artery (RCA). Additionally, as far as we researched, this is the first surgical case report of AAORCA with an aberrant right subclavian artery (RSCA).

## Case presentation

A 17 year-old man reported a cardiac arrest during a basketball game. Therefore, AED detected ventricular fibrillation, and he was defibrillated accordingly. The spontaneous circulation subsequently returned, and he was transferred to our hospital. When the patient reached our hospital, his blood pressure was 95/48 mmHg and heart rate was 98/min sinus rhythm, and he had no particular symptoms other than complaints of thirst. He had no medical history or family history of cardiac disease.

Later, according to the cardiac catheterization, the left coronary artery (LCA) was intact, but the RCA could not be selectively imaged. Additionally, the acetylcholine (ACh) stress test failed to detect the spasm in LCA.

**Fig. 1:**
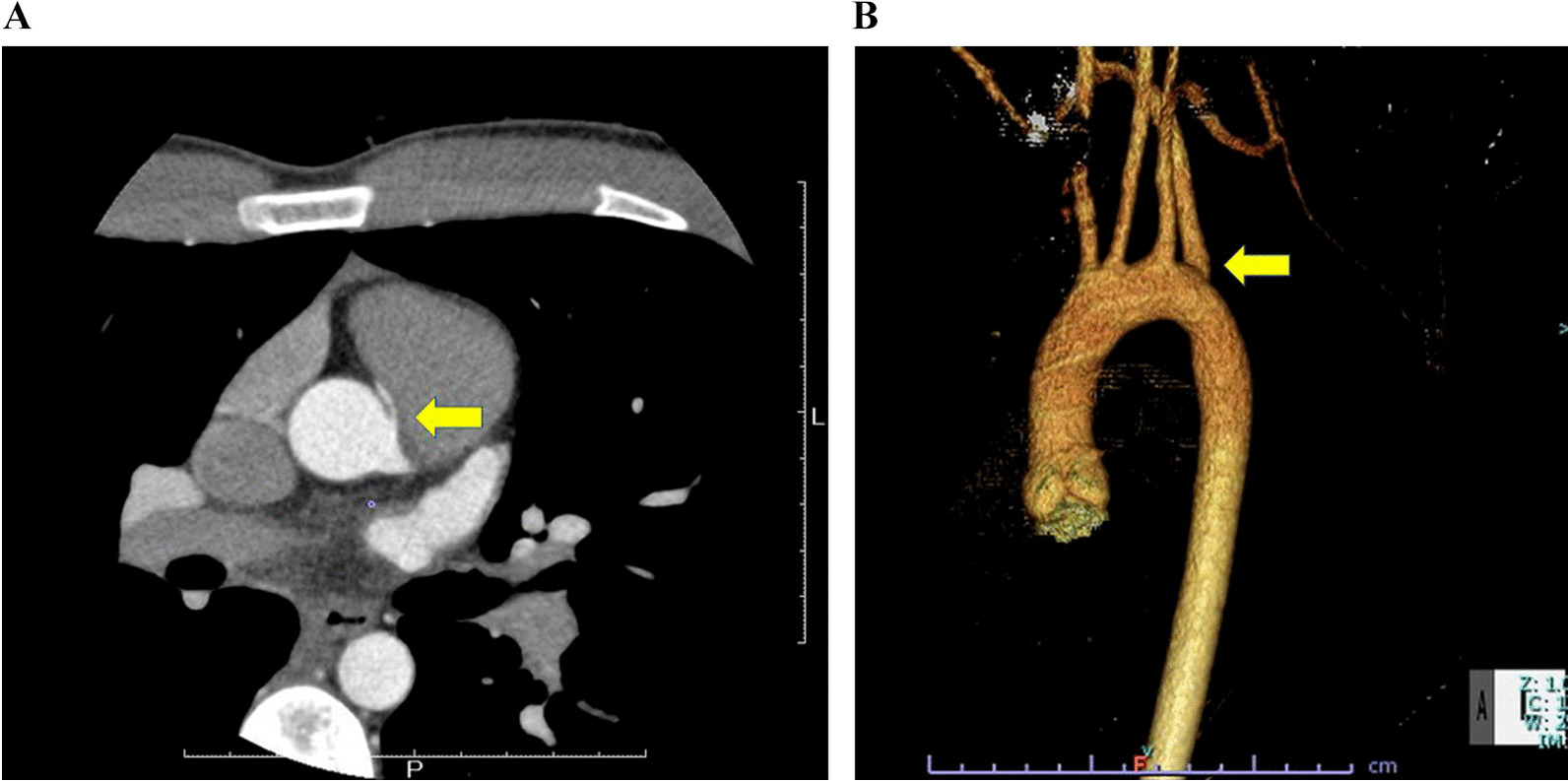
**A** Coronary CT (computed tomography) revealed anomalous aortic origin of a coronary artery. **B** Three-dimensional CT revealed an aberrant right subclavian artery

Ischemia could not be demonstrated using exercise stress myocardial scintigraphy up to 200 W overload. Conversely, the electrophysiological study did not induce any arrhythmia. However, coronary computed tomography (CT) revealed the anomalous aortic origin of the RCA from the left Valsalva sinus (Fig. 1A). RCA had stenosis between the aortic and pulmonary arteries along with a slit-like orifice from the Valsalva sinus. An aberrant RSCA was detected in a CT scan (Fig. 1B). Nonetheless, echocardiography confirmed his intramural RCA and patent foramen ovale (PFO).

After various detailed examinations, we performed a reimplantation of the RCA.

Cardiopulmonary bypass was established using median sternotomy with systemic perfusion from ascending aortic and bicaval drainage. Under the beating heart, the RCA was dissected. The direct-aortic echography determined the position of the aortic leaflet commissure. And we also marked the anastomotic sites. Later, antegrade and retrograde cardioplegia induced cardiac arrest. RCA was double ligated at the exiting part from the aorta; thus, RCA transection was done. Then, we made an anastomosis hole with a 3.5 mm puncher and performed the anastomosis with 7 − 0 polypropylene thread. PFO closure was also performed as a concomitant procedure.

**Fig. 2:**
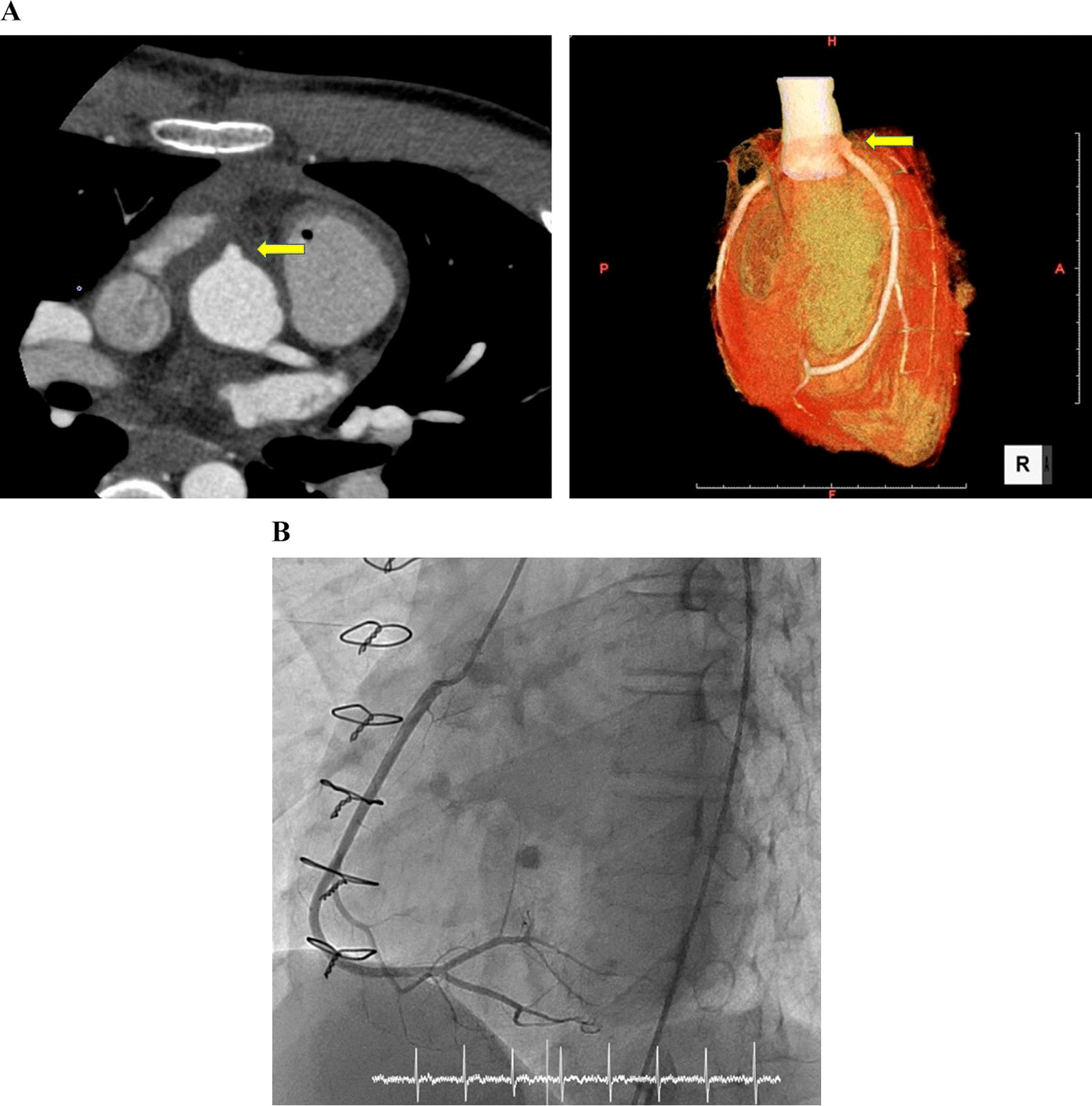
**A** Postoperative Coronary CT (computed tomography) revealed that anastomosis of the right coronary artery to the aorta was good. (Arrows indicate the anastomosis site. Right: axial image, Left: 3-D image). **B** Postoperative coronary angiography 3 month after operation

Postoperative coronary CT confirmed that the anastomosis site was good (Fig. 2A). Later, the patient was discharged on postoperative day 10.

Since the preoperative imaging of the RCA was insufficient, and the coronary artery spasm induction test was not performed preoperatively, a follow-up coronary angiography was performed 3 months after the operation. The results confirmed that there are no problems with the anastomotic site (Fig. 2B). Further induction of coronary spasm (acetylcholine loading) revealed negative results.

## Discussion and conclusion

There are many reports on AAORCA treatments, such as unroofing and coronary artery bypass grafting. However, the number of cases is small, and there are a few reports about long-term outcomes.

In the case of intramural RCA, unroofing may require a procedure to peel off the commissure, which may cause aortic valve regurgitation eventually. It may also be necessary to add pulmonary artery translocation for interarterial RCA [3]. According to the preoperative assessment, we did not select unroofing because RCA might have been an intramural type running behind the commissure.

Coronary artery bypass grafting may require ligation on the proximal portion of RCA due to the risk of flow competition, and graft patency eventually could be an issue in younger cases.

Reimplantation is technically simple, and the lesion is easily approachable, even if a stenotic lesion in RCA occurs in the future. Previous cases also showed that it might enable surgeons to avoid aortotomy [2, 4]. Although, depending on the diameter of the RCA in early childhood, reimplantation could be difficult in principle, it should be considered as an option for adolescent cases with AAORCA. Previous studies that reported on more than 10 cases [1, 2] suggested that anastomosis could be performed if the proximal part of the RCA had a diameter of 3.5 mm because a 3.5 mm puncher was often used to create neo-ostium in the ascending aorta.

The main purpose was to evaluate the distance up to which the proximal part of the RCA could be detached to mobilize it for anastomosis without tension. Detaching the proximal part of the RCA until the first conal branch could be essential [1, 2]. We thought that the assessment of preoperative CT was helpful.

Although there are a few reports on reimplantation, the report by Amadou et al. [2] shows promising results in the mid-term follow-up of 7 years. Thus, it could be an effective method after adolescence.

In this study, there was an aberrant RSCA from the descending aorta. To our knowledge, this is the first surgical report of the AAORCA with aberrant RSCA. If a coronary artery disease might occur in the future period after coronary artery bypass graft with the right internal thoracic artery (ITA) to the RCA, percutaneous coronary intervention could be more difficult through the right ITA. Reimplantation was a good treatment option in this case.

## Data Availability

The data underlying this article will be shared on reasonable request to the corresponding author.

## Abbreviations

AAORCA: Anomalous aortic origin of the right coronary artery
RCA: Right coronary artery
RSCA: Right subclavian artery
AED: Automated external defibrillator
LCA: Left coronary artery
Ach: Acetylcholine
CT: Computed tomography
PFO: Patent foramen ovale
